# Answering human papillomavirus vaccine concerns; a matter of science and time

**DOI:** 10.1186/1750-9378-8-22

**Published:** 2013-06-12

**Authors:** David Hawkes, Candice E Lea, Matthew J Berryman

**Affiliations:** 1 The Florey Institute of Neuroscience and Mental Health, The University of Melbourne, Victoria 3010, Australia; 2 Research Centre for Injury Studies, Flinders University, Bedford Park, South Australia 5042, Australia; 3 SMART Infrastructure Facility, University of Wollongong, New South Wales 2522, Australia

**Keywords:** Human papillomavirus, HPV, Safety, Cervical cancer, Cancer prevention, Adverse events, Clinical trials

## Abstract

Since the introduction of the HPV vaccine, questions have been asked about its efficacy in preventing cancer linked with HPV. Concerns about the HPV vaccine safety profile have also been raised. This paper highlights the rapidly growing body of evidence (including clinical trials and post-marketing surveillance) illustrating both the safety of the HPV vaccine, through a detailed investigation of reported adverse events, and its efficacy in reducing both HPV infections rates and the resulting drop in cervical lesions, which have been demonstrated to be good predictors of cervical cancer risk.

## Background

The first human papillomavirus (HPV) vaccine, Gardasil®, was registered in Australia in 2006 and was followed in 2009 by Cervarix®. However, since the introduction of these HPV vaccines both their safety and efficacy have been questioned [1]. These include valid questions such as whether these vaccines will reduce vaccine-associated HPV infection rates, how long vaccination will provide protection for and the role natural exposure could play, and whether a compensatory increase in non-vaccine HPV infection will be observed or if the vaccine will provide some degree of cross-protection. While there were some data available when these vaccines were introduced it is not possible to observe the effects of any medical intervention at a population level before its introduction. According to the manufacturers of Gardasil® and Cervarix®, over 120 million doses of these two HPV vaccines have been distributed globally, with over 200 studies involving human clinical trials and post-market surveillance undertaken and published. This review will examine the questions raised about the effectiveness and safety of the HPV vaccine and how they have been, and are, being addressed by the scientific/medical research community. It is important to note that there are a number of concerns about HPV vaccines, and indeed vaccination in general, which are of a more social, economical or political nature, such as whether people are given appropriate levels of information prior to vaccination, and merit a more in-depth discussion however they fall outside the scope of this review.

### Does vaccination against HPV prevent infection with HPV?

When examining any vaccine the primary question is: does it reduce the impact of the targeted pathogen, either through reducing infection itself or minimising the clinical effects of infection? In the case of HPV vaccines; Gardasil®, a quadrivalent vaccine, targets HPV types 6, 11, 16, and 18 and Cervarix®, a bivalent vaccine, targets HPV types 16 and 18. Studies (described in Table 1) have demonstrated that the HPV vaccine is able to reduce the infection rate of vaccine-associated HPV types (HPV 16/18) by over 90% [2,3] in HPV naïve women and this reduction is maintained for at least 5 years [4]. A rather elegant demonstration of how exposure to HPV increases the efficacy of vaccination is described by Herrero and colleagues [5] who looked at the rate of protection against the vaccine associated HPV 16/18 at different timepoints after vaccination. They showed that at 22 months post HPV vaccination the vaccine was 71% efficacious, by 34 months the efficacy is up to 92% and beyond 46 months it is 100%, in the group of participants who had all three doses of Cervarix® and had a negative test for at least one of the vaccine types (HPV16 or HPV18). Clinical trials often present data for a number of subpopulations, but the most relevant for HPV vaccination is the group that is HPV negative prior to vaccination but may not get all three doses of the vaccine. This group represents the most realistic model of the population who receive the vaccine, pre-teen (so unlikely to have been HPV exposed) but may not receive all three doses over 6 months. This population has been defined as either modified intention to treat- (MITT) or total vaccine cohort- (TVC) naïve [6]. The other group is the TVC [5,7] or intention to treat (ITT) [8,9] and includes all participants in the trial irrespective of how many doses they receive and over what time period, whether they have had prior HPV exposure, and so on. The major outcome that large scale clinical trials of vaccines examined was the rate of pre-cancerous lesions, such as cervical intraepithelial neoplasia (CIN, grades 1, 2, or 3 and above) or adenocarcinoma *in situ* (AIS) associated with HPV (reviewed in [6]). Vaccination demonstrated high efficacy against the HPV16/18 associated CIN2 (approx. 99-100%), CIN3 (approx. 100%) and AIS (approx. 100%) in MITT/TVC-naïve subpopulations (Table 2). Vaccination also provided high protection for the TVC group, which includes individuals previously exposed to HPV, against HPV16/18 type associated CIN2 (>54.8%), CIN3 (>45.1%) and AIS (>60%) [7,8,10] (Table 2). HPV vaccination is highly efficient at reducing both HPV 16/18 and associated pre-cancerous lesions, particularly when given to a HPV naïve population such as that targeted by mass vaccination programs.

**Table 1:** Characteristics of phase III efficacy studies in young women including end of study cohort numbers

**Vaccine**	**FutureI Gardasil®**	**Future II Gardasil®**	**PATRICIA Cervarix®**
No. study sites	62	90	135
Countries included	16	13	14
Length of trials (years)	4	4	4
Control	225 μg Aluminium hydroxyphosphate sulphate	225 μg Aluminium hydroxyphosphate sulphate	Hepatitis A Vaccine (including AS04)
Age (years)	16-24	16-26	15-25
Primary endpoints	Incident HPV6/11/16/18-associated genital warts, CIN1-3, VIN1-3, ValN1-3, AIS and cervical, vaginal or vulvar cancer	Incident HPV 16/18-associated CIN2-3, AIS or cervical cancer	Incident HPV 16/18-associated CIN2+
No. in ITT/TVC-naive cohort	4618-4689		5466
No. in ITT/TVC-naive cohort control	4680-4735		5452
No. In ITT/TVC	8562		8694
No. In ITT/TVC control	8598		8708

Abbreviations: *AIS* Adenocarcinoma *in situ*, *CIN* cervical interepithelial neoplasia, *VIN/VaIN* Vulvar/vaginal intraepithelial neoplasia. FUTURE I/II study number of subjects varies depending on endpoint or HPV type under analysis. Adapted from [6].

**Table 2:** **HPV vaccine efficacy against genital disease in FUTURE I**/**II** (**Gardasil**®) **and** PATRICIA **(Cervarix®) trials**

**HPV 16/18 associated**	**% efficacy (95% CI)**	**Rate reduction**
**FutureI/II**		
*MITT/TVC-naive*		
CIN2	100.0 (91.9-100)	0.3
CIN3	100.0 (90.5-100)	0.2
AIS	100.0 (<0-100)	<0.1
VIN2/3 or VAIN2/3	95.4 (71.5-99.9)	<0.1
Genital warts	96.4 (91.4-98.9)	0.8
ITT/TVC		
CIN2	54.8 (40.8-65.7)	0.3
CIN3	45.1 (29.8-57.3)	0.3
AIS	60.0 (<0-87.3)	<0.1
VIN2/3 or VAIN2/3	78.5 (55.2-90.8)	<0.1
Genital warts	79.5 (73.0-84.6)	0.8
**PATRICIA**		
*MITT/TVC-naive*		
CIN2+	99.0 (94.2-100)	0.47
CIN3+	100.0 (85.5-100)	0.13
AIS	100.0 (15.5-100)	0.03
*ITT/TVC*		
CIN2	60.7 (49.6-69.5)	0.43
CIN3	45.7 (22.9-62.2)	0.13
AIS	70.0 (–16.6-94.7)	0.02

Abbreviations: *AIS* Adenocarcinoma *in situ*, *CIN* cervical interepithelial neoplasia, *VIN/VaIN* Vulvar/vaginal intraepithelial neoplasia, *MITT* modified intention to treat, *ITT* Intention to treat, *TVC* Total vaccine cohort, *CI* Confidence interval. Rate reduction is measured as per 100 women years. Adapted from [6].

### Will HPV vaccination cause a compensatory rise in non-vaccine HPV types?

There are more than 100 HPV types but only 15 have been classed as being a high risk to progress from infection to cancer (oncogenic); 16, 18, 31, 33, 35, 39, 45, 51, 52, 56, 58, 59, 68, 73, and 82 [11]. Obviously HPV16/18 are targeted by vaccination but there have been questions asked as to whether a decrease in HPV16/18 will be counterbalanced by an increase in non-vaccine oncogenic HPV types and even a possible overall increase in cancer.

Schiller and colleagues [6] reviewed the results of the large scale clinical trials of both HPV vaccines (FUTURE I/II [12], PATRICIA [7] and Costa Rica HPV Vaccine Trial [13]). They examined the rates of 6 month persistent infection of 12 non-vaccine HPV types and found that both vaccines provided significant protection against oncogenic HPV types similar to HPV16, (39, 45, 59, and 68). Both vaccines also provided significant protection against HPV31; in addition Cervarix® significantly reduced rates of HPV33 and 52. While the duration of vaccine coverage (95% protection) for HPV16/18 has been demonstrated to remain for at least 5 years [4,14,15], long term trials for the duration of cross-type protection are currently not available.

As outlined previously, one of the major outcomes examined by clinical trials of these vaccines is the prevalence of HPV positive AIS and CIN lesions. The PATRICIA study [7] found Cervarix® provided cross-protective efficacy against four non-vaccine oncogenic HPV types (31, 33, 45, and 55) measured by persistent infection and CIN2+ lesion rates. When CIN2+ and CIN3+ lesion rates, associated with the composite findings of 12 non-vaccine oncogenic HPV types (31, 33, 35, 39, 45, 51, 52, 56, 58, 59, 66, and 68) were examined, the vaccinated group showed reduced incidence in both HPV naïve (56% [CIN2+] and 91% [CIN3+]) and TVC (34% and 47%) groups compared with the non-vaccinated.

Other studies also provide evidence for vaccine cross-protection against non-vaccine HPV types in a variety of conditions, such as reduced HPV35 [16] infection rates in Finnish adolescents four years post-vaccination, and the production of cross-reactive antibodies against HPV31 [17] in HIV positive children. Joura and colleagues [18] examined data from the FUTURE I/II trial and found women vaccinated with Gardasil® after they had undergone surgery for cervical disease or were diagnosed with vulvar or vaginal disease (genital warts, vulvar intraepithelial neoplasia, or vaginal intraepithelial neoplasia), had lower rates of CIN (1+, 2+ or 3+), genital warts, and vulvar (or vaginal) intraepithelial neoplasia (1+ or 2+).

While both vaccines are very effective against HPV16/18 and appear to provide cross-protection against some non-vaccine oncogenic HPV types it is worth specifically investigating whether vaccination is actually causing a net reduction in HPV associated AIS and CIN lesions. As Cervarix® had better cross-protective properties it is not surprising that the TVC displayed larger reductions is all HPV associated markers; CIN2+ (33.1%), CIN3+ (45.6%) and AIS (76.9%) [7]. However, Gardasil® still demonstrated reductions in CIN2 (19.3%), CIN3 (16.4%) and AIS (62.5%) rates compared with the non-vaccinated cohort [8,10]. It should also be noted that when only HPV naïve individuals were analysed, much higher protection from CIN2+ (64.9), CIN3+ (93.2) and AIS (100%) was observed [7]. These data show that vaccination is reducing the pathological signs of all HPV type infections, particularly in HPV naïve individuals.

### Will vaccination against HPV prevent (cervical) cancer?

One of the most frequently raised concerns about HPV vaccination is that one of the major outcomes of clinical trials, cervical intraepithelial neoplasia (CIN) are not good predictors for progression to cervical cancer, thus making it impossible to say on that basis alone whether HPV vaccines will reduce cervical cancer incidence. It is worth taking a moment to investigate if firstly there is a link between HPV and CIN in the first place. As described above, clinical trials have demonstrated HPV vaccination reduces CIN lesion incidence. This is not surprising, as a systematic review and meta analysis of over forty trials, and 22,000 women, found that, although there was a lot of variation in methodology, a persistent HPV infection was “consistently and strongly associated” with CIN2/3 lesions [19]. It has also recently been shown that the average time from initial HPV infection to the appearance of cervical lesions is 43–50 months (~4 years) [20]. The literature overwhelmingly demonstrates that HPV is a, if not the, major cause of cervical lesions such as CIN2 and CIN3. It was also demonstrated as far back as 1976 that untreated CIN3 lesions result in cervical cancer 28–39% of the time [21]. A review of over 40 years of published studies determined that the likelihood of progression of CIN1 to cancer was 1%, for CIN2 it was 5% and for CIN3 greater than 12% [22].

A recent study demonstrated the importance of HPV as a determinant of (pre)invasive cervical cancer when they showed that 3.7% of the women, in a study of over 330,000 women, with normal cervical cytology (pap smear) and positive HPV status experienced 34% of the CIN3+, 29% of the cancers and 63% of the adenocarcinomas [23]. There is also other evidence in the literature to support this finding, specifically that HPV vaccinated individuals have lower rates of CIN2+ and CIN3+ [1,4] and that HPV vaccine types significantly correlate with progression from CIN2+ to CIN3+ [24].

In terms of a biological mechanism we know that certain HPV types are strongly associated with different chromosomal changes, in particular those associated with sections of DNA containing tumor-suppressing genes [25,26]. These changes are in turn strongly associated with cervical cancer [25,27]. Although the development of cancer is complex [28], the pathway variable from person to person [29], and not every persistent HPV infection progresses to cancer [29], a number of papers have even examined the absolute risk of cervical cancer from HPV infection [30,31]. Overall, HPV can be associated with 99.7% of cervical cancers and can be considered as a necessary cause of cervical cancer [27], even though not all HPV infections progress to CIN, and then to cancer. It should be noted that while this paper is primarily focussed on cervical cancer, HPV infection is also associated with cancers of the penis (40% HPV associated), vulvar/vaginal (40%), anal (90%), mouth (3%) and oropharynx (12%) [32]. In addition the Gardasil® vaccine targets two non-oncogenic types 6 and 11 which are a leading cause of genital warts. A recent Australian study found a significant (P<0.001) decrease in diagnosis of genital warts in women under 30 years of age [33]. This age group is the first to be vaccinated against HPV and these decreases in genital warts were not seen in older age groups.

### Is HPV vaccination safe?

Since introduction, safety concerns have been raised about reported serious adverse reactions to HPV vaccination. A number of these concerns are about vaccine ingredients in general but the safety of these ingredients has been well established (reviewed in [34]). It is worth noting that the Cervarix® vaccine includes the Adjuvant System 04 (AS04) which combines 3-0-desacyl-4’-monophosphoryl lipid A (MPL) and aluminium salt to increase the immune response to vaccination. Verstraeten and colleagues [35] reviewed the use of AS04 in vaccines (68, 512 participants) to determine whether its use could cause an increase in autoimmune diseases. They determined that there was no increase in relative risk (RR) of experiencing an autoimmune event compared with a control group that containing non-adjuvanted, or aluminium-/aluminium hydroxide-adjuvanted vaccines (RR 0.98, confidence intervals 0.8, 1.21). An examination of Gardasil® safety studies [36] revealed that the vaccine produced significantly higher rates of injection site adverse events (82.9%) than the aluminium containing placebo (77.4%) which in turn produced significantly higher rates than the saline placebo (49.5%). This is an expected outcome, as described above aluminium containing adjuvants stimulate the immune system. However when systemic adverse events were examined there was no difference between vaccine and placebo. The rest of the review will focus on the safety profile of HPV vaccines as a whole, rather than examining individual constituents.

Adverse events have been reported following HPV vaccination (Table 3) but clinical trial data demonstrates that there is no difference in the rate of serious adverse events between the either HPV vaccine and controls (RR 1.00, 95% CI 0.91 – 1.09). A study examining the adverse events reported following Gardasil® vaccination found that the overwhelming majority (>94%) of these reactions are minor and are largely local injection site reactions (for example redness, swelling, pain at injection site) but do include other minor self limiting reactions such as syncope (fainting episodes), headache and nausea (reviewed in [37]). Similar data for Cervarix® vaccination does not appear to have been reported as yet.

**Table 3:** Serious adverse events following HPV vaccination

**Study**	**% vaccine**	**% control**	**Relative risk (95% CI)**
Future I	1.8	1.7	1.07 (0.71-1.60)
Future II	0.7	0.9	0.83 (0.56-1.24)
Harper	4.1	3.5	1.17 (0.64-2.14)
Koutsky	0.3	0.3	1.34 (0.30-5.96)
Munoz	0.2	0.4	0.43 (0.11-1.65)
Villa	0.7	0.7	1.01 (0.14-7.10)
*Patricia*	7.5	7.5	1.00 (0.91-1.11)
**Total**			1.00 (0.91-1.09)

Adapted from [6,38]. Original trial data from FUTURE I/II [12], Harper, Koutsky [39], Munoz [3,40], Villa [41], and PATRICIA [7].

Evidence from large scale clinical trials has been utilised to assess whether serious adverse events are more likely post HPV vaccination with a systematic review and meta-analysis having been undertaken to examine the combined results of 7 unique randomized clinical trials (including the previously mentioned FUTUREI/II and PATRICIA trials) of HPV vaccines [38]. These 7 trials included over 44,000 women. When the authors examined whether vaccination was associated with serious adverse events, they found that the chance of having a serious adverse event was identical whether the individual was vaccinated or in the control group. Even when the trials were looked at individually there was still no difference in adverse event incidence between the vaccinated and control populations.

Large scale clinical trials can provide information on adverse events prior to mass vaccination but their statistical power is limited by their participant numbers. The clinical trials described above included over 44,000 women and as such may not be expected to reliably detect rare (e.g. less than 1 in 100,000) adverse events. Passive reporting systems such as the U.S. post-marketing safety surveillance program database VAERS can provide information that can help identify (rare) adverse events. As VAERS is an open system where any member of the general public can enter a vaccine reaction it is difficult to directly analyze the publically available data to assess causal relationship between notified events and vaccine administration without further investigation. Gold and colleagues [42] give an interesting example of the peculiarities of passive reporting systems, focusing on the Australian context. In 2009 the adverse event reporting rate for Gardasil® was 24 per 100,000 but the reporting rate for exactly the same vaccine in the USA was 53.9 per 100,000. There are a number of possible causes for these differences in adverse event rates such as uneven denominators, ease of reporting, public knowledge of the reporting system or even cultural/religious/political reasons, and these variables provide yet another example of the importance of follow-up investigations of adverse event reports.

A number of examinations of the VAERS data for HPV vaccination have shown a low rate of adverse reactions, and no link for any causal relationship between HPV vaccination and reports [43,44]. There are a variety of conditions that can occur in the absence of HPV or other vaccinations, in young adolescent females, which can be mistaken for HPV vaccination side-effects, and therefore to draw conclusions from adverse event data to HPV vaccination is to mistake (time) correlation for causality [45]. In 2009 Slade and colleagues [44] investigated the 32 deaths attributed to Gardasil® that had been reported on VAERS. Of the 32 deaths there was not enough information to identify or verify the death for 12 reports. The causes of the remaining 20 deaths were: 2 due to diabetic ketoacidosis, 3 due to pulmonary embolism, 6 were cardiac-related (4 arrhythmias, 2 myocarditis), 2 were idiopathic seizure disorders, 4 were unexplained, 1 was due to juvenile amyotrophic lateral sclerosis, 1 case of *Neisseria meningitidis* serogroup B caused meningoencephalitis and the final death was related to prescription drug abuse. The authors concluded that statistically (proportional reporting ratio of 1.2 for 8- to 29-year olds) these results were not significantly (p=0.92) different from what you would expect from a similar sized unvaccinated population.

A recent study by an Australian group [42] systematically examined adverse events in the first years (2007 – 2009) of the HPV vaccination program during which time over 5.8 million doses of Gardasil® were distributed nationally. They found 1394 suspected adverse events were reported using a passive surveillance program. One possible severe side effect of the HPV vaccine may be an increased rate of anaphylaxis but as with much data from passive reporting systems it is not definitive. New South Wales reported a rate of 2.6 per 100,000 vaccines compared with a rate of 0.5 per 100,000 in South Australia and Victoria combined. Gold and colleagues present a number of possible reasons for this including the older age of recipients, different mechanisms of surveillance and a number of other causes for this discrepancy. Only 12 cases were reported during the timeframe investigated so it is difficult to know whether this is a vaccine induced event until more information becomes available.

It has also been suggested that HPV vaccination can increase the probability of progression of established persistent infection to CIN2+ or higher. This was based on the report of a single small study [46], however further analysis yielded evidence that the vaccinated cohort had higher (pre-vaccination) risk factors than the placebo group. The authors were concerned about the effect of the biased risk factor profile and small numbers on the data so further analysis was undertaken by pooling data from three studies (including the risk factor biased study). This larger data set showed no difference in CIN2+ or higher presentation between the vaccinated and placebo cohorts.

Other specific concerns about serious adverse events following HPV vaccination such as the possibility of increased autoimmune conditions in the vaccinated, increased incidence of Guillain-Barré Syndrome (reviewed in [44]) or increased severe adverse events caused by interactions with other vaccines have so far been proven unfounded [47,48].

## Conclusions

The first HPV vaccine was introduced in 2006 and since its introduction it has been a topic of controversy, with a number of questions being asked about the vaccine; Did it work? How long would the protection last? Would there be an increase in HPV types not covered by the vaccine? Did it actually prevent cancer? Was it safe?

In the seven years since the registration of the first HPV vaccine these and many other questions have been investigated by the scientific/medical research community. This review describes a large number of studies that have analysed the growing set of safety data, and have demonstrated the safety of HPV vaccines and answered the very specific concerns raised, particularly in regards to nervous system reactions, interactions with other vaccines, and HPV vaccine influencing the course of existing lesions. In terms of virology the current evidence shows that HPV vaccination is highly efficient at preventing vaccine associated HPV types and that protection is well over 90% if given to HPV naïve individuals [2-4]. Additionally it appears that HPV vaccination may even also offer some cross-protection against the 13 non-vaccine oncogenic HPV types, including HPV31, 33, 35, 39, 45, 52, 59, and 68 [4,6,16]. The longevity of the HPV vaccination has also been investigated and there are currently studies that demonstrate immunogenicity lasts at least 5 years for both Gardasil® and Cervarix® [12,14-16].

HPV vaccination has been introduced for less than 7 years and as such it is difficult to quantitate the effect it will have on the incidence of cervical, vulvar/vaginal, penile, anal and other cancers. There is very strong, some say conclusive data, that HPV is the root cause of over 99% of cervical cancers [22-24,27]. HPV vaccination has been clearly demonstrated to reduce the incidence of the pre-cancerous markers of cervical cancer, in trials involving over 44,000 women [6], and the resulting effects on cervical cancer incidence will become clearer over time with the aid of post-marketing surveillance. A recent meta-analysis of HPV testing has concluded that HPV testing provides an advantage over equivocal cytologic screening for CIN with the added benefit that genotyping for HPV16 and HPV18 assists medical professionals in better assessing HPV-associated risk [49]. Australia is uniquely positioned to be a world leader in monitoring the efficacy and safety of HPV vaccines at a population level due to its early adoption of Gardasil® as evidenced by the distribution of over 5.8 million doses by 2009 [42].

The goal of this review was to investigate the published scientific/medical literature to determine whether the oft repeated queries about HPV vaccination safety and efficacy have been examined. The rapidly growing body of research, including immunology, virology, public health, epidemiology and a number of other fields, can allow the whole community including doctors, medical researchers, parents and other interest groups to be more confident that the benefits of HPV vaccination far outweigh the risks and that mechanisms are in place to continue monitoring possible adverse events into the future.

## Abbreviations

HPV: Human papillomavirus; CIN: Cervical intraepithelial neoplasia; MITT: Modified intention to treat; ITT: Intention to treat; TVC: Total vaccine cohort; AIS: Adenocarcinoma *in situ* .

## Competing interests

The authors have no competing interests and received no funding for this work.

## Authors’ contributions

DH chose the topic and wrote and researched the initial version of the review. CEL and MJB were involved in the literature research and contributed to the writing and drafting of the manuscript. All authors have read and approved the final manuscript.
